# Activity limitation and participation restriction in Osteoarthritis and Rheumatoid arthritis: findings based on the National Health and Nutritional Examination Survey

**DOI:** 10.1186/s12891-022-05607-z

**Published:** 2022-07-06

**Authors:** John Marwa Gikaro, Hao Xiong, Feng Lin

**Affiliations:** 1 Department of Rehabilitation Medicine, Sir Run Run Hospital, Nanjing Medical University, Nanjing, People’s Republic of China; 2 Faculty of Rehabilitation Medicine, Kilimanjaro Christian Medical University College, P.O. Box 2240, Moshi, Kilimanjaro Tanzania; 3 School of Rehabilitation Medicine, Nanjing Medical University, No.140, Hanzhong Road, Gulou District, Nanjing, Jiangsu Province People’s Republic of China

**Keywords:** Osteoarthritis, Rheumatoid arthritis, Activity limitation, Participation restriction, Physical functioning, International Classification of Functioning, Disability and Health (ICF).

## Abstract

**Background:**

Osteoarthritis (OA) and Rheumatoid arthritis (RA) are the most common joint diseases leading to chronic pain and disability. Given the chronicity and disabling nature of OA and RA, they are likely to influence full participation of individuals in the society. An activity limitation occurs when a person has difficulty executing an activity; a participation restriction is experienced when a person has difficulty participating in a real-life situation. The aim of this study was to examine the associations between OA and RA and the domains of activity limitation and participation restriction.

**Methods:**

A cross-sectional study design comprised 3604 adults from the 2009 to 2018 National Health and Nutrition Examination Survey (NHANES). All participants aged ≥ 20 years with complete data were included. Activity limitation and participation restriction were assessed by reported difficulty in performing 14 tasks selected from Physical Functioning Questionnaire. Data on OA and RA were obtained from Medical Conditions Questionnaire. Weighted logistic regression model was used to examine the associations between OA and RA and the selected tasks.

**Results:**

Over 36% of participants had limitations. Both OA (OR = 2.11) and RA (OR = 2.36) were positively associated with activity limitation and participation restriction (*p* < 0.001). Poor or fair health was associated with difficulty in physical functioning, with highest odds observed in leisure activities (OR = 2.05), followed by difficulty in attending social events (OR = 1.99), walking for a quarter mile (OR = 1.97), preparing meals (OR = 1.93) and walking up ten steps (OR = 1.92).

**Conclusion:**

Adults with OA and RA had nearly similar odds of having activity limitations and participation restrictions. Difficulty in executing most activities of daily living (ADLs) has significant association with poor or fair health. Holistic interdisciplinary care to individuals with OA or RA focusing on ADLs and environmental factors may improve health status.

## Background

Osteoarthritis (OA), the most common joint disease in the world [1, 2], is a degenerative joint disease that involve the joint cartilage and other intra-articular structures [3]. It affects both joints of the axial and appendicular skeleton; such as, the knees, hips, spinal facets and sacroiliac, as well as joints of the hand and foot [4]. OA leads to pain, stiffness, reduced joint function [5] and ultimately disability [6]. On the other hand, Rheumatoid arthritis (RA) is the most common inflammatory joint disease among rheumatic diseases. It is estimated to affect 1% of the population worldwide. The disease presents with pain, stiffness and symmetrical swelling of peripheral joints [7]. If left untreated, RA can cause irreversible joint damage, deformities, disability [8] chronic pain, reduced health-related quality of life (HRQoL) [7], and higher mortality [9].

Physical functioning is described as the ability to perform basic movements and more complex activities [10]. These activities are considered essential for maintaining independence in fulfilling usual roles in daily life. Limitations in physical functioning leads to declined body physical functions, activity dependence and reduced HRQoL [11]. Moreover, constraints in physical functioning is associated with long term risk of disability [12]. As both OA and RA lead to chronic pain and disability, which impede physical functioning, it is critical to address their impact on the ability to perform certain activities of daily living (ADLs), and participate in life situation or socio-economic events.

The World Health Organization (WHO) officially endorsed the International Classification of Functioning, Disability and Health (ICF) in 2001. The ICF is a classification of health and health-related domains that provides a standard language for describing and measuring health and disability. Besides health and functioning, this description enables experts to assess activities and environmental factors that influence full participation of individuals in the society. According to the ICF: activity is described as the execution of a task or action by an individual; and participation is termed as a person’s involvement in a life situation [13]. The most accepted clinical assessment tools for arthritic conditions are: the Western Ontario and McMaster Universities Osteoarthritis Index (WOMAC) —for assessment of patient-relevant treatment effects in OA [14]; and the American College of Rheumatology (ACR) response criteria and the European League Against Rheumatism (EULAR) response criteria —for assessment of patient-relevant treatment effects in RA [15]. In both cases, they incorporate some domains of activity limitation and participation restriction presented in the ICF checklist. However, much of the recent researches on activity limitation and participation restriction in individuals with OA or RA has been focusing on specified body-part limitations after particular arthritic condition [16–18], hence little is known on the limitations existing in individuals with OA and RA, comparatively. Similarly, previous studies used few clinical samples with limited generalizability. In attempt to fill in the existing gaps, our study used a large, nationally representative sample of the United States (U.S.) adults over a period of 10 years comparing the overall activity limitation and participation restriction in individuals with OA and RA.

Therefore, the aim of the present study was to examine the associations between two categories of arthritis (OA and RA) and the domains of activity limitation and participation restriction assessed in self-reported physical functioning questionnaire of NHANES among U.S. adults. Further, we also wanted to explore the association between domains of limitations and the perception of general health condition. Findings from our study may be useful as baseline data for promoting functional independence and improving self-rated health status among patients with OA and RA.

## Methods

### Study design and participants

In this study, we used the 2009 to 2018 NHANES, a two-year cycle population-based survey designed by the U.S. National Center of Health Statistics (NCHS) to assess and monitor the health and nutrition of the U.S. household population. The survey uses a complex multistage sampling method to recruit a nationally representative sample that estimates health conditions of the U.S. inhabitants. Both home interview and physical examination are conducted by highly trained medical personnel. All methods were carried out in accordance with relevant ethical principles of the Declaration of Helsinki, and the administration etiquette was ratified by the NCHS Research Ethics Review Board. All participants eligible for the study provided written informed consent. Our final sample included only adults thereby excluding children or adolescents under the age of 20 years.

### Covariates

Computer-assisted personal interviewing (CAPI) approach was used to collect demographics information. The respondent selected English or Spanish as the language of interview, or requested for an interpreter. A wide range of information was collected, such as: age, gender, ethnicity (assigned as Mexican American, other Hispanic, non-Hispanic white, non-Hispanic black, or other race), education level (categorized as less than 9th grade, 9 – 11th grade, high school, some college or associate degrees and college graduates or above) and family poverty income ratio (PIR). Body mass index (BMI) was established by measuring height in standing position, in centimeters, and body weight in kilograms.

To account for health problems that may cause difficulty in executing or participating in the selected tasks, the following comorbidities were considered: asthma, congestive heart failure, coronary heart disease, cancer, diabetes, depression, stroke and smoking status [11, 19]. Survey participants were asked to report on health problems, one by one, from the listed secondary health conditions in medical conditions questionnaire, or separate questionnaires for diabetes and mental health – depression and smoking status. Each question had four responses: “Yes,” “No,” Refused” and “Don’t know,” and responses were regarded “Missing” if they did not fall under such categories. In each category of comorbidities, we included affirmative responses for respective conditions thus remaining with a binary variable i.e., “Yes” and “No,” only. In asthma, for example, the question was: ‘The following questions are about different medical conditions. Has a doctor or other health professional ever told you that you have asthma (az-ma)?’ Further information regarding the NHANES 2009–2018 Questionnaire Instruments can be found within each data cycle at the Centers for Disease Control and Prevention (CDC) website [20].

### Arthritis types

Data on OA (or degenerative arthritis) and RA were obtained from “Medical Conditions Questionnaire”. The questionnaire provided self-reported personal interview data on a broad range of health conditions in which respondents were asked, ‘Has a doctor or other health professional ever told you that you had arthritis?’ This was followed by another question: ‘Which type of arthritis was it?’ Participants had to answer ‘Yes’, ‘No,’ ‘Refused’ or ‘Don’t know’ in all items, but those who reported ‘No’ in the first question did not have to answer the second question. Participants answered ‘Yes’ if they had been told by a doctor or other health professional that they had OA or RA of any joint. Only affirmative answers were considered hence the rest were dropped. Participants with or without deformities were allowed to answer the questionnaire.

### Activity limitation and participation restriction

Our main outcome variables were obtained from the “Physical Functioning Questionnaire” that provided self-reported data on functional limitations. In this study, we selected 14 activities (Table 1) that represent the components of activity limitation and participation restriction of the ICF [13]. Participants were asked to report the level of difficulty that they had in performing these tasks by themselves and without using any special equipment. Each item used had four affirmative levels of responses, i.e., no difficulty, some difficulty, much difficulty and unable to do. Those who did not do the activity, refused to answer, or responded not to know whether they do the activity were considered missing thus dropped from the master file. To identify those having limitations, we coded the responses no difficulty = 0, some difficulty = 1, much difficulty = 2 and unable to do = 3, and then recorded the sum of all responses for each individual record. The total sum ranged between 0 and 42. Participants with 0 score were considered having no limitations and those who had score of 1 or higher were regarded as with limitations in executing or participating in at least one activity.

**Table 1:** List of activities included in the analysis for determining activity limitation and participation restriction

Selected activities
Attending social event difficulty
Dressing yourself difficulty
Getting in and out of bed difficulty
Going out to movies, events difficulty
Grasp/holding small objects difficulty
Leisure activity at home difficulty
Lifting or carrying difficulty
House chore difficulty
Preparing meals difficulty
Standing from armless chair difficulty
Standing for long periods difficulty
Sitting for long periods difficulty
Walking for a quarter mile difficulty
Walking up ten steps difficulty

### Data management and statistical analysis

Data files from different health component measurement questionnaires within each cycle were linked with participant identification number (variable name: SEQN) according to data user guidelines provided by NHANES. In order to ensure that 10-year data is matched appropriately, variables from each 2-year cycle were merged using ‘SEQN’ after renaming corresponding variables in each cycle. After combining data from all 5 cycles, a 2009–2018 master file was created. Missing data at random coded as ‘.’ and the data from the non-affirmative respondents coded as ‘7 s’ or ‘9 s’ (referring to ‘Refused’ and ‘Don’t know’) in all questionnaires including demographics questionnaire were dropped from the master file.

For descriptive analysis, Mean and standard deviation (SD) for normally distributed continuous variables, median and interquartile range (IQR) for normally distributed continuous variables, and weighted percentages for categorical variables were calculated. For inferential statistics, Odds ratios and their value of 95% confidence intervals from logistic regression model were used to determine the association between activity limitation and participation restrictions and OA and RA. Three models were performed as follows: model 1, unadjusted; model 2, adjusted for demographic characteristics, i.e., age, gender, race, education level, BMI and family income ratio; and model 3, adjusted for demographic characteristics in model 2, smoking and other comorbidities such as, asthma, congestive heart failure, coronary heart disease, stroke, cancer, diabetes and depression. We also assessed the association between specific activity limitation and self-rated health condition. The model was fitted for three categories of general health condition, i.e., ‘excellent health,’ ‘very good or good health’ and ‘fair or poor health’ that was obtained from “Current Health Status Questionnaire,” taking ‘very good or good’ as reference. Statistical significance was set at *P* < 0.05 in all models.

All the data management and analyses were conducted using R software for Windows (version 4.1.0). The analysis followed the analytic guidelines on the NHANES website. The sample weights were used to account for the complex sampling methods used during data collection so as to avoid biased estimates for the U.S. population.

## Results

A total of 49,693 survey participants completed the 2009 to 2018 NHANES survey. Activity limitation and participation restriction was assessed only in adults with osteoarthritis or degenerative arthritis, and rheumatoid arthritis. After excluding children or adolescents under the age of 20 years, the sample remained 27,705. Out of 27,705 individuals, 3604 participants that is representative of 147.5 million adults in the U.S. completed all details of all questionnaires used in this study.

### Demographic characteristics

Majority of participants were females, and over 60 years old. More than 36% of participants had difficulties in executing certain activities or participating in certain life occasions, those with OA leading. The proportion of participants having OA was over five times higher than with RA. The detailed participant characteristics is given in Table 2.

**Table 2:** Participant demographic characteristics stratified by difficulty in executing or participating in an activity

Variables	Overall	Have difficulty	No difficulty
	*N* = 3604	*N* = 1274	*N* = 2330
	N(%)	%	%
Gender = Female	1833(54.7)	64.0	49.4
Age (median [IQR])	67.00 [62.00, 73.00]	68.00 [62.00, 75.00]	66.00 [62.00, 72.00]
Age group (years)			
20–39	129(5.2)	8.3	3.4
40–59	121(5.8)	10.6	3.2
60–79	2934(80.2)	68.4	86.9
80 or above	420(8.8)	12.7	6.5
Ethnicity/race			
Mexican American	390(3.5)	4.1	3.2
Other Hispanic	341(2.9)	2.3	3.3
Non-Hispanic White	1754(81.1)	81.4	80.9
Non-Hispanic Black	720(7.1)	7.1	7.0
Other Race	399(5.4)	5.1	5.6
Education			
< 9th grade	332(3.7)	4.1	3.4
9-11th grade	392(7.0)	7.9	6.6
High school	780(21.3)	22.8	20.4
College or University	2100(68.0)	65.3	69.6
BMI (mean (SD))	29(6)	30(7)	28(5)
BMI group			
Underweight	45(1.2)	1.2	1.2
Normal	949(26.2)	23.1	28.0
Overweight	1374(36.9)	31.0	40.3
Obese	1236(35.7)	44.7	30.5
General health condition			
Excellent	391(13.1)	6.6	16.8
Good or very Good	2674(77.9)	78.6	77.5
Fair or Poor	539(9)	14.8	5.8
Arthritis type			
No arthritis	2598(66.9)	54.4	74.1
Osteoarthritis	765(27.9)	37.9	22.1
Rheumatoid arthritis	241(5.2)	7.7	3.8
Depression^a^ = Yes	601(16.6)	22.2	13.4
Smoking = No	1983(55.3)	53.6	56.2

All percentages shown are weighted

*Abbreviations:* *IQR* Interquartile Range, *SD* Standard Deviation

^a^ Having a feeling of little interest or pleasure in doing things

### Association of arthritis type and activity limitation and participation restriction

Unadjusted and adjusted models showed significant association between OA and RA and activity limitation and participation restriction (*p* = 0.000). In a fully adjusted model (model 3), OA (OR = 2.11) and RA (OR = 2.36) were positively associated with activity limitation and participation restriction. The comparison of all models showed that study participants were more than 2 times likely of having limitations regardless of the type of arthritis (Table 3).

**Table 3:** Association between OA and RA and presence of activity limitation and participation restriction

Type of arthritis	Activity limitation and participation restriction	
	OR (95% CI)	*P* -value
Model 1^a^		
No arthritis	Ref	
Osteoarthritis	2.33 (1.87–2.91)	0.000
Rheumatoid arthritis	2.74 (1.85–4.04)	0.000
Model 2^b^		
No arthritis	Ref	
Osteoarthritis	2.17 (1.70–2.78)	0.000
Rheumatoid arthritis	2.55 (1.73–3.74)	0.000
Model 3^c^		
No arthritis	Ref	
Osteoarthritis	2.11 (1.64–2.71)	0.000
Rheumatoid arthritis	2.36 (1.60–3.46)	0.000

*Abbreviations:* *Ref* Reference category, *OR* Odds Ratio, *CI* Confidence Interval

^a^ Unadjusted model

^b^ Adjusted for demographic characteristics (age, gender, race, education level, BMI, income)

^c^ Adjusted for demographic characteristics (age, gender, race, education level, BMI, income), smoking, comorbidities (asthma, congestive heart failure, coronary heart disease, stroke, cancer, diabetes, depression)

### Association between activity limitations and current health status rating

There was significant association between perceived health status and activity limitation and participation restrictions. Poor or fair health was positively associated with situations such as difficulty in performing leisure time activities, attending social events, walking for a quarter mile, walking up to ten steps, preparing meals, standing for long periods, sitting for long periods, lifting or carrying things, and going out to movies. Excellent health, however, was positively associated with difficulty in dressing, and getting in and out of bed. The odds ratios and respective confidence intervals are shown in Table 4.

**Table 4:** Association between activity limitations and self-rated current health status

Activity	General health condition		
	Good or very good	Poor of fair	Excellent health
	OR (95% CI)	OR (95% CI)	OR (95% CI)
Attending social event difficulty	Ref	1.99 (0.68–0.69)	0.69 (0.36–0.37)
Dressing yourself difficulty	Ref	1.31 (0.27–0.28)	1.60 (0.47–0.48)
Getting in and out of bed difficulty	Ref	0.68 (0.38–0.39)	1.45 (0.37–0.38)
Going out to movies, events difficulty	Ref	1.02 (0.01–0.02)	0.45 (0.80–0.81)
Grasp/holding small objects difficulty	Ref	0.61 (0.49–0.50)	0.59 (0.53–0.54)
Leisure activity at home difficulty	Ref	2.05 (0.71–0.72)	0.06 (2.87–2.91)
Lifting or carrying difficulty	Ref	1.07 (0.06–0.07)	0.87 (0.13–0.14)
House chore difficulty	Ref	0.91 (0.09–1.00)	0.35 (1.05–1.06)
Preparing meals difficulty	Ref	1.93 (0.65–0.66)	0.00 (394.27–394.27)
Standing from armless chair difficulty	Ref	0.67 (0.39–0.40)	0.62 (0.47–0.48)
Standing for long periods difficulty	Ref	1.45 (0.36–0.37)	0.66 (0.40–0.41)
Sitting for long periods difficulty	Ref	1.15 (0.13–0.14)	0.97 (0.02–0.03)
Walking for a quarter mile difficulty	Ref	1.97 (0.68–0.69)	0.49 (0.71–0.72)
Walking up ten steps difficulty	Ref	1.92 (0.65–0.66)	0.37 (0.99–1.00)

*Abbreviations:* *CI* Confidence Interval, *OR* Odds Ratio, *Ref* Reference category

*p*-value = 0.000 in all components

## Discussion

There have been tremendous increase of research and clinical interests towards activity limitation and participation restriction since the introduction of ICF by WHO over 20 years ago, particularly in rehabilitation medicine. Arthritic conditions, being one of disabling conditions, are likely to cause psychological, physiological or anatomical impairments. In this study we assessed the association of having activity limitation and participation restriction related to OA and RA in a nationally representative sample of non-institutionalized adults. To the author’s knowledge, this is the first large-scale, 10-year population-based study to evaluate this relationship. We found that OA was more prevalent than RA as reported in previous study [21]. Like in previous studies, we found that majority of individuals with OA or RA were older than 60 years [22–26]. Further, one-third of participants had limitations in activity and participation. Both OA and RA showed higher statistical significance level of associations with almost all domains of activity limitation and participation restriction, and self-reported health status.

Our findings showed that approximately 45% of the study participants with either of the arthritis reported having difficulties in executing one or more activities of daily life or in participating in life situations. This finding was analogous to a previous study by Barbour et. al., which found that approximately 43% of individuals with arthritis experienced arthritis-attributable activity limitations [27]. A possible explanation to such a high proportion of activity limitation among adults with arthritis could be the body parts affected by particular arthritis. For example, RA mainly target peripheral joints [7] and OA attack both larger and smaller joints [4], which in both cases result into pain and deformities. Therefore, individuals suffering from either of these arthritic conditions are likely to avoid some activities because of present deformity that hamper effective execution of intended action, or fear of provoking the pain. Another possible reason for this similarity could be because most individuals with arthritis seemingly presents with similar clinical features, hence comparable effects. Likewise, findings from the current study showed that nearly two-thirds of those with limitations were females. This observation is also consistent with previous researches [28–30] because arthritis has been linked to increasing age and female sex. Therefore, inasmuch as more than half of study participants were females, it was more likely that higher proportion of females suffered from the effects of either of the arthritis [31].

Participant characteristics showed that nearly half of survey participants with obesity were encountering more difficulties in participating in life situation or executing at least one activity. This finding can be explained by evidence from a previous study that showed an association of obesity and greater arthritis activity [32]. Moreover, obesity is associated with reduced physical activity and overall physical functioning. Also, those who reported poor or fair health condition also reported having activity limitations and participation restrictions by almost three folds compared with those with no difficulties. This finding correlates with findings from previous study that shows that activity limitation was associated with old age and poor health condition [33]. One possible explanation for our finding is that people with activity limitation tend to have reduced self-esteem and eventually rate themselves with poor health status as they cannot perform most ADLs. Nevertheless, our findings showed that non-Hispanic whites had higher prevalence of arthritis-attributable activity limitations than among non-Hispanic blacks, which was contrary to previous findings [27]. This difference can be explained by higher representation of non-Hispanic whites in this study.

According to our results, both unadjusted (crude) and adjusted models showed consistent significant association of OA and RA with activity limitation and participation restriction. In both cases, the odds ratios were greater than 2 (*p* < 0.05), suggesting that adults with OA or RA are highly likely to have difficulty in participating in life situations or activities. Although there are limited studies showing a comparable association in both arthritis, a review by Lo et. al., reported higher associations of back pain and arthritis (OA) with greater effects on activity and work [21]. This shows that individuals with arthritis suffer economic impact probably because of lost ability of engaging in productive activities. This finding of nearly equal levels of activity limitation and participation restriction in both individuals with OA and RA could be explained by the concept from previous studies showing that people with musculoskeletal manifestations report having several limitations in the daily life due to a mismatch between the capabilities of the person, the demands of the environment and the requirements of the activities [34].

Adults with poor or fair general health condition reported limitations in multiple activities and participation compared with those with good or very good health conditions. Similar findings were previously observed in older inactive persons [35]. A possible reason behind this finding could be linked with reduced HRQoL as activity limitations, other medical conditions and social factors affect the individual’s quality of life [36]. Another possible explanation for this finding is that rheumatic diseases such as RA, causes fear in the general population, especially due to associated joint deformities and subsequent disabilities [37], as a result majority feel insecure in terms of physical, emotional and social well-being. Poor/fair health condition was highly reported by individuals with difficulties in attending social events, going out to movies, performing leisure activities, lifting or carrying things, preparing meals, standing for long periods difficulty, sitting for long periods difficulty, walking for a quarter mile difficulty and walking up ten steps difficulty (*p* < 0.05); of which the highest odds were at leisure activity at home difficulty followed by attending social event and walking for a quarter mile. This finding corresponds to a previous study that observed higher participation restriction in sports/recreation/leisure/cultural activity, and difficulties in standing for long periods and walking long distances [38]. Physiological processes with aging may explain this situation as adults with advanced age become frail [39], therefore, an addition of any disabling condition or comorbidity could affect physical functioning and overall health condition of an individual.

One big strength of our study is using a large, 10-year nationally representative sample. Hence, generalizability of results can be drawn owing to diverse sociodemographic and topographic characteristics of study participants. However, our findings had several limitations. First, NHANES data are based on self-reported questionnaires, making them subject to misclassification of responses. Nevertheless, according to our findings most adults recruited were literate, thus able to provide reliable answers. Second, as the NHANES is cross-sectional, the associations that we observed might not be causal and might have resulted from other potential confounders that we did not adjust for. In adult research, for example, arthritic conditions may co-occur with other cardiometabolic comorbidities, such as heart diseases and diabetes mellitus [40], which in turn also affects physical functioning. However, we accounted for adjusting measured comorbidities in attempt to establish the relationships stated. Third, not all domains of activity limitation and participation restriction, according to the ICF, were assessed in the NHANES physical functioning questionnaire. Fourth, selection of heterogeneous patients may have caused the difference between OA and RA not to be revealed. Despite these limitations, adults with OA or RA in our analysis reported significant activity limitation in ADLs and life events.

## Conclusions

Adults with OA reported having significant activity limitation and participation restriction at almost similar odds of those with RA. Findings showed significant association of poor or fair health status with difficulties in performing leisure time activities, walking, standing and sitting for long periods, lifting or carrying things and participation in social or outing events. We advocate holistic interdisciplinary care to individuals with OA or RA that focuses on activities of daily living and environmental factors so as to promote physical functioning and improve the general health status.

## Data Availability

The datasets generated and/or analyzed during the current study are available in the NHANES repository, [https://wwwn.cdc.gov/nchs/nhanes/Default.aspx].

## Abbreviations

ACR: The American College of Rheumatology
ADLs: Activities of daily living
BMI: Body mass index
CAPI: Computer-assisted personal interviewing
CDC: The Centers for Disease Control and Prevention
EULAR: The European League Against Rheumatism
HRQoL: Health-related quality of life
ICF: International Classification of Functioning, Disability and Health
IQR: Interquartile range
NCHS: National Center of Health Statistics
NHANES: National Health and Nutrition Examination Survey
OA: Osteoarthritis
OR: Odds ratio
PIR: Poverty income ratio
RA: Rheumatoid arthritis
SD: Standard deviation
U.S.: United States
WHO: World Health Organization
WOMAC: The Western Ontario and McMaster Universities Osteoarthritis Index
